# Evaluation of Anticancer and Antibacterial Activity of Four 4-Thiazolidinone-Based Derivatives

**DOI:** 10.3390/molecules27030894

**Published:** 2022-01-28

**Authors:** Bartosz Skóra, Anna Lewińska, Anna Kryshchyshyn-Dylevych, Danylo Kaminskyy, Roman Lesyk, Konrad A. Szychowski

**Affiliations:** 1 Department of Biotechnology and Cell Biology, Medical College, University of Information Technology and Management in Rzeszow, Sucharskiego 2, 35-225 Rzeszow, Poland; rlesyk@wsiz.edu.pl (R.L.); kszychowski@wsiz.edu.pl (K.A.S.); 2 Department of Biotechnology, Institute of Biology and Biotechnology, College of Natural Sciences, University of Rzeszow, Pigonia 1, 35-310 Rzeszow, Poland; alewinska@ur.edu.pl; 3 Department of Pharmaceutical, Organic and Bioorganic Chemistry, Danylo Halytsky Lviv National Medical University, Pekarska 69, 79010 Lviv, Ukraine; kryshchyshyn.a@gmail.com (A.K.-D.); dankaminskyy@gmail.com (D.K.)

**Keywords:** anticancer properties, heterocycles, cell cycle, cytotoxicity, synthetic compounds

## Abstract

Heterocycles are commonly known for their unique features, e.g., antibacterial or anticancer properties. Although many synthetic heterocycles, such as 4-thiazolidinone (4-TZD), have been synthesized, their potential applications have not yet been fully investigated. However, many researchers have reported relevant results that can be a basis for the search for new potential drugs. Therefore, the aim of this study was to evaluate the cytotoxic, cytostatic, and antibacterial effects of certain 4-thiazolidinone-based derivatives, Les-3166, Les-5935, Les-6009, and Les-6166, on human fibroblasts (BJ), neuroblastoma (SH-SY5Y), epithelial lung carcinoma (A549), and colorectal adenocarcinoma (CACO-2) cell lines in vitro. All tested compounds applied in a concentration range from 10 to 100 µM were able to decrease metabolic activity in the BJ, A549, and SH-SY5Y cell lines. However, the action of Les-3166 was mainly based on the ROS-independent pathway, similarly to Les-6009. In turn, Les-5935 and Les-6166 were able to promote ROS production in BJ, A549, and SH-SY5Y cells, compared to the control. Les-3166, Les-6009, and Les-6166 significantly increased the caspase-3 activity, especially at the concentrations of 50 µM and 100 µM. However, Les-5935 did not induce apoptosis. Only Les-5935 showed a minor cytostatic effect on SH-SY5Y cells. Additionally, the antibacterial properties of the tested compounds against *P. aeruginosa* bacterial biofilm can be ranked as follows: Les-3166 > Les-5935 > Les-6009. Les-6166 did not show any anti-biofilm activity. In summary, the study showed that Les-5935, Les-6009, and Les-6166 were characterized by anticancer properties, especially in the human lung cancer cell. In cases of BJ, SH-SY5Y, and CACO-2 cells the anticancer usage of such compounds is limited due to effect visible only at 50 and 100 µM.

## 1. Introduction

Small molecules based on different heterocyclic building blocks have been widely used in medicine, food industry, and biology [1]. The presence of noncarbon atoms in the heterocyclic rings is responsible for their unique features. Due to the wide diversity of the structures of heterocycles, their physical and chemical properties differ substantially [2]. However, these properties do not fully illustrate/predict their biological effects in the cell; therefore, it is necessary to evaluate different heterocycle subtypes and consider their impact on biological systems. Heterocyclic compounds also occur in the environment as part of plant secondary metabolites [3,4] and show various effects, including in vitro cytotoxicity towards some cancer cell lines [5]. Many other heterocyclic compounds with high biological importance, such as serotonin, histidine, etc., are spread in the human organism. Nowadays, heterocycles are still of special interest in medicinal chemistry and the development of new drug-like molecules [1,6,7,8].

One of the well-known groups of heterocycles are 4-thiazolidinones (4-TZD), which are considered to be promising compounds as privileged scaffolds for new drug design [9,10,11]. A variety of biological effects was shown for 4-thiazolidinone-based compounds, and plenty of lead compounds, drug candidates, and approved drugs were found in this group [12,13,14]. With their chemical features, 4-thiazolidinones are widely and successfully applied in different approaches for drug discovery. Molecular hybridization or the so-called hybrid pharmacophore approach has been one of the efficient approaches in the search for novel hit compounds. This method consists of combining several privileged heterocyclic cores/pharmacophoric units into one single molecule, yielding new chemical entities with new biological properties [15,16,17,18]. A series of 5-ene-4-thiazolidinones seem to be interesting as compounds possessing low toxicity along with several types of pharmacological activity (e.g., antimicrobial and antitumor) [12,19,20]. The latter are considered as hit compounds for further optimization in order to increase the selectivity/specificity of action [21]. 4-Thiazolidinones are also efficient building blocks in the substructure-based diversity-oriented synthesis approach [22], which yields related biologically active heterocyclic derivatives (4-TZD-based compounds), among which thiopyrano[2,3-*d*]thiazoles and their polycyclic analogues are of special interest [23]. Moreover, 4-thiazolidinones can be treated as precursors for highly active molecules with a related structure via in vivo metabolic transformation [24]. In all cases, the design of anticancer and antimicrobial agents based on a 4-thiazolidinone core is a challenging and important task.

It was reported that *rel*-*N*-(2,4-dichlorophenyl)-2-[(5aR,11bR)-2-oxo-5a,11b-dihydro-2*H*,5*H*-chromeno[4′,3′:4,5]thiopyrano[2,3-*d*][1,3]thiazol-3(6*H*)-yl]acetamide (Les-2194), 5,10-dihydro-2*H*-benzo[6,7]thiochromeno[2,3-*d*][1,3]thiazole-2,5,10-trione (Les-3377), and 3-{2-[5-(4-dimethylaminophenyl)-3-phenyl-4,5-dihydropyrazol-1-yl]-4-oxo-4,5-dihydro-1,3-thiazol-5-ylidene}-2,3-dihydro-1*H*-indol-2-one (Les-3640) exerted a high toxic effect on human squamous carcinoma cells (SCC-15). They were found to act mainly in a PPARγ-dependent manner [25,26]. A similar effect was produced by the action of 5*Z*-(4-fluorobenzylidene)-2-(4-hydroxyphenylamino)-thiazol-4-one (Les-236), e.g., in lung carcinoma (A549) and colon adenocarcinoma (CACO-2) cell lines and in normal skin fibroblasts (BJ) [27]. However, 4-TZD-based compounds are also able to have a negative influence on healthy cells, such as mouse preadipocytes (3T3-L1 cell line), although this effect is observed only at high micromolar concentrations (100 µM of Les-2194 and Les-3640) [28].

Moreover, many 4-TZDs have shown antibacterial properties, as they reduce bacterial viability efficiently through different mechanisms [12,13,29,30]. Interestingly, the antimicrobial agent halicin (5-[(5-nitro-1,3-thiazol-2-yl)sulfanyl]-1,3,4-thiadiazol-2-amine) [31], which is the first compound newly discovered with the help of artificial intelligence, also contains a thiazole frame in its structure and can be regarded as an example of a drug-repurposing strategy [32,33]. However, it is generally difficult to predict biological effects of heterocycles on normal, cancer, or microbial cells. Therefore, it is necessary to perform screening research to show the effects of newly obtained heterocycles at the cell level.

**Figure 1:**
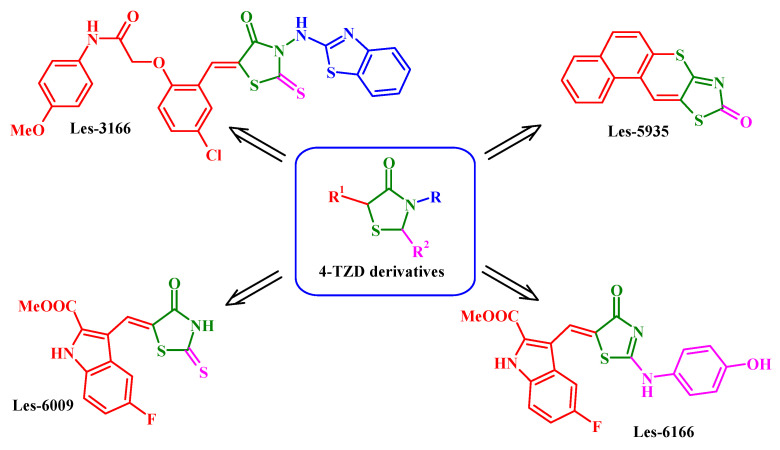
Structure of studied compounds.

The four 4-TZD-based compounds: 2-{2-[3-(benzothiazol-2-ylamino)-4-oxo-2-thioxothiazolidin-5-ylidenemethyl]-4-chlorophenoxy}-N-(4-methoxyphenyl)-acetamide (Les-3166), 7-oxa-10-thia-8-aza-cyclopenta[*b*]phenanthren-9-one (Les-5935), 5-fluoro-3-[2-(4-hydroxyphenylamino)-4-oxo-4*H*-thiazol-5-ylidenemethyl]-1*H*-indole-2-carboxylic acid methyl ester (Les-6166), and 5-fluoro-3-(4-oxo-2-thioxothiazolidin-5-ylidenemethyl)-1*H*-indole-2-carboxylic acid methyl ester (Les-6009) (Figure 1) were synthesized as potent drug-like molecules. The structure rationales for Les-3166, Les-6009, and Les-6166 design are based on the crucial role of the C-5 substituent of the 4-thiazolidinone core for biological activity (e.g., anticancer activity) realization. The advantage of this approach is that most hit and lead compounds belong to the 5-ene-4-thiazolidinones [12,34]. Moreover, our previous structure activity relationship (SAR) analysis showed that except for the C-5 fragment diversity, the creation of hybrid molecules via combination of TZD core with other “pharmacologically attractive” fragments (indole, benzothiazole, etc.) is also the prospective direction of TZD-based molecular design [12,18,35]. The chemical approach to Les-5935 is based on our hypothesis that 5-ene-4-thiazolidinone fixation in a fused thiopyrano[2,3-*d*]thiazole ring allows conservation of the activity vector, which opens new possibilities of molecule optimization. Thus, fused thiopyrano[2,3-*d*]thiazoles could be of special interest as cyclic mimetics of their pharmacologically attractive precursors [23]. The anticancer potential of mentioned derivatives has not been fully investigated. Moreover, Les-3166, Les-5935, Les-6009, and Les-61166 have been designed as the potential ligands of PPARγ, which is able to regulate the cell proliferation and/or intracellular oxidative stress response [36]. Therefore, the aim of this study was to evaluate the effect of these compounds on cancer cell lines in contrast to healthy cells by determination of their impact on metabolic activity, reactive oxygen species (ROS) generation, and apoptosis induction. The impact of the tested compounds on cell cycle progression was also evaluated. Moreover, their potential antibacterial properties were determined using a bacterial strain that is able to form an antibiotic-resistant biofilm.

## 2. Results

### 2.1. Impact of 4-Thiazolidinone Derivatives on the Metabolic Activity

**Figure 2:**
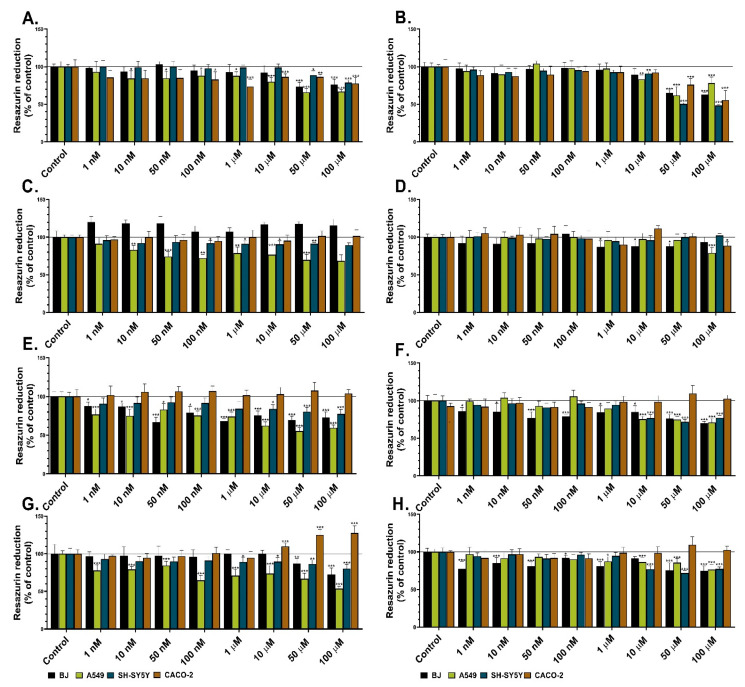
Metabolic activity of BJ, A549, SH-SY5Y, and CACO-2 cell lines after 24 h (**A**,**C**,**E**,**G**) and 48 h treatments (**B**,**D**,**F**,**H**) with Les-3166 (**A**,**B**), Les-5935 (**C**,**D**), Les-6009 (**E**,**F**), and Les-6166 (**G**,**H**). Mean values (*n* = 6) with standard deviation (error bars) where *, **, and *** are statistically different from the respective (vehicle-treated) control at *p* < 0.05, *p* < 0.01, and *p* < 0.001, respectively (one-way ANOVA, Tukey’s test).

The resazurin reduction assay was used in this study to evaluate the influence of certain heterocycles (Les-3166, Les-5935, Les-6009, and Les-6166) on the metabolic activity in four cell lines. Les-3166 significantly decreased the metabolic activity of BJ only at the concentrations of 50 and 100 µM after both the 24 h and 48 h treatments (by 27% and 24% at the 24 h exposure and by 35% and 38% at the 48 h exposure, respectively, compared to the control) (Figure 2A,B). Les-3166 decreased the metabolic activity in the A549 cell line in the range of 10 nM–100 µM after 24 h (from 16% to 34%, compared to the control). However, after the 48 h treatment, the decrease was observed only at the concentrations of 10–100 µM (17% to 39%, compared to the control) (Figure 2A,B). The SH-SY5Y cell line treated with Les-3166 for 24 h showed a decrease in metabolic activity only at the concentrations of 50 and 100 µM (12% and 22%, respectively, compared to the control). After the next 48 h of treatment of SH-SY5Y, a decrease was observed for 10, 50, and 100 µM (from 10% to 52%, in comparison to the control) (Figure 2A,B). The effect of Les-3166 on the CACO-2 cells was noticed at 100 nM–100 µM after 24 h (a 10–27% decrease in the cell metabolism, compared to the control). However, after 48 h, the metabolic activity was significantly reduced only at the concentrations of 50 and 100 µM (by 24% and 45%, respectively, compared to the control) (Figure 2A,B).

Les-5935 did not cause significant reduction of metabolic activity in the normal human fibroblasts (BJ) after the 24 h treatment (Figure 2C). However, after the 48 h treatment, the tested compound reduced the metabolic activity of the BJ cells only at the 1 µM–100 µM concentrations (ranging from 13% to 14%, compared to the control) (Figure 2D). In the A549 cell line, loss of metabolic activity was observed after the 24 h Les-5935 treatment in all concentrations but 100 nM (up to 32%, compared to the control). After the 48 h treatment, a significant decrease in metabolic activity was observed only at the concentration of 100 µM (Figure 2D). In the SH-SY5Y cell line exposed to the action of Les-5935, the metabolic activity was decreased only in the concentration range of 100 nM–100 µM (from 9% to 12%, in comparison to the control) (Figure 2C). After 48 h, Les-5935 showed no impact on neuroblastoma cell line. (Figure 2D). Les-5935 did not show any effect on the metabolic activity of the CACO-2 cell line after the 24 h exposure. However, after 48 h, Les-5935 decreased the metabolic activity of the CACO-2 cells by 12%, compared to the control, at the 100 µM concentration (Figure 2C,D).

Les-6009 caused a significant decrease in the metabolic activity of the BJ cells at all tested concentrations after the 24 h treatment (from 13% to 31%, compared to the control) (Figure 2E). A similar effect was observed after 48 h, i.e., Les-6009 caused reduction of cell metabolism at all tested concentrations in the BJ cell line (ranging from 24% to 30%, compared to the control) (Figure 2F). Les-6009 decreased the metabolic activity of the A549 cells at all tested concentrations, ranging from 24% to 45%, compared to the control. After the 48 h treatment, the cell metabolism was decreased only in the concentration range from 10 to 100 µM (up to 29%, compared to the control) (Figure 2E,F). In the SH-SY5Y cell line, the decrease in metabolic activity was observed after the 24 h and 48 h treatments in the concentration range from 10 to 100 µM (up to 29%, compared to the control) (Figure 2E,F). In turn, after the 24 h and 48 h treatments with Les-6009, the CACO-2 cell line did not show any relevant changes in metabolic activity (Figure 2E,F).

Les-6166 significantly decreased the metabolic activity of the BJ cells only at 50 and 100 µM after the 24 h contact with the cells. However, the 48 h treatment with this compound in the concentration range between 50 nM and 100 µM caused a decrease in the BJ metabolic activity (from 15% to 26%, compared to the control) (Figure 2G,H). After 24 h of exposure of the A549 cells to Les-6166, a 16%–47% reduction of metabolic activity was observed at all tested concentrations. In turn, toxic effects were detected after the 48 h treatment only at the 10, 50, and 100 µM concentrations (up to 24%, compared to the control) (Figure 2G,H). Moreover, toxic effects of the tested compound on the SH-SY5Y cells were observed after the 24 h and 48 h treatments at the concentrations of 1, 10, 50, and 100 µM (after the 24 h exposure) and 10, 50, and 100 µM (after the 48 h exposure) (Figure 2G,H). Interestingly, Les-6166 did not decrease the metabolic activity of the CACO-2 cells at either of the time intervals (Figure 2G,H).

### 2.2. Impact of 4-Thiazolidinone Derivatives on ROS Production

**Figure 3:**
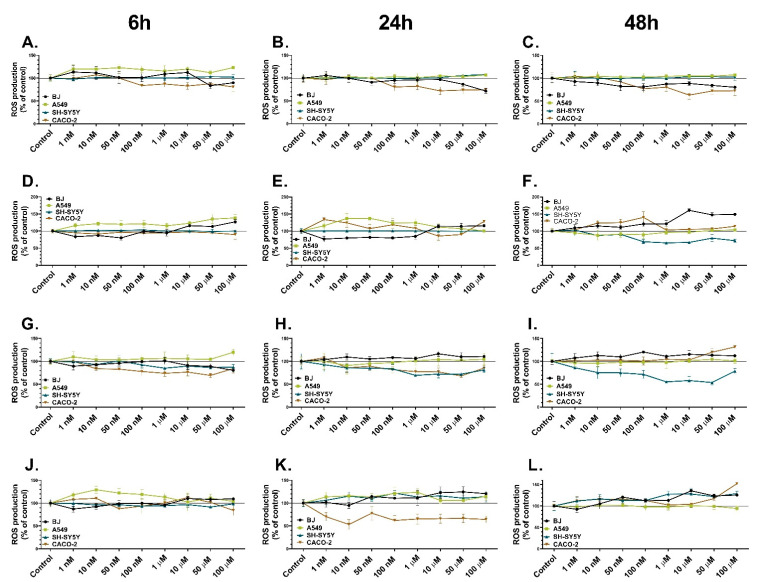
ROS production in BJ, A549, SH-SY5Y, and CACO-2 cell lines after treatment with increasing concentrations of Les-3166 (**A**–**C**), Les-5935 (**D**–**F**), Les-6009 (**G**–**I**), and Les-6166 (**J**–**L**) after 6 h (**A**,**D**,**G**,**J**), 24 h (**B**,**E**,**H**,**K**), and 48 h (**C**,**F**,**I**,**L**). The statistical significance of each data point analyzed using one-way ANOVA is described in the Supplementary Data (Tables S1–S12).

The H_2_DCF-DA probe was used in this study to assess the impact of the tested substances on ROS generation in normal (BJ) and cancer (A549, SH-SY5Y, and CACO-2) cell lines. Les-3166 caused a notable increase (up to 13%) in ROS production in the BJ cells only after the 6 h treatment in the concentration range from 1 nM to 10 µM, compared to the control (Figure 3A). However, after the 24 h and 48 h treatments, Les-3166 reduced the ROS level in the BJ cell line at all tested concentrations (from 10% to 21%, compared to the control) (Figure 3B,C). A549 cells treated with Les-3166 in the concentration range from 1 nM to 100 µM for 6 h showed a significant increase in ROS production (up to 23%, compared to the control). However, no significant changes in ROS production were observed after the 24 h and 48 h treatments (Figure 3A–C). Moreover, Les-3166 was not able to induce any changes in ROS generation in the SH-SY5Y cells at any time intervals (Figure 3A–C). CACO-2 cells treated with Les-3166 in the concentration range from 100 nM to 100 µM for 6 h exhibited reduced ROS production (up to 20%, compared to the control). The same tendency was observed after the 24 h and 48 h treatments (Figure 3A–C).

Les-5935 applied at the concentrations of 10, 50, and 100 µM induced a significant increase in ROS generation just after 6 h in the BJ cells (from 15% to 27%, compared to the control). A similar tendency was noticed after the 24 h and 48 h treatments with Les-5935 (Figure 3D–F). Les-5935 at all concentrations caused a high increase in ROS production in the A549 cells (from 16% to 38%, compared to the control) after 6 h. However, after the 24 h treatment, this tendency was observed only in the concentration range of 1 nM–1 µM; in turn, no significant changes in ROS production were detected after the 48 h treatment (Figure 3D–F). Les-5935 at any concentration had no impact on the SH-SY5Y cells after 6 h, similarly to the 24 h treatment. The 48 h treatment with 10 nM–100 µM of Les-5935 resulted in a decline in ROS production (up to 35%, compared to the control) (Figure 3D–F). The CACO-2 cells treated with Les-5935 for 6 h did not show any changes in ROS production. However, after the 24 h treatment, increased ROS production (up to 43%, compared to the control) was observed in the concentration range from 1 nM to 1 µM; this tendency was noted after the 48 h treatment with Les-5935 in the same concentrations (Figure 3D–F).

Les-6009 did not cause any significant changes in ROS production in the BJ cells after the 6 h exposure. After the 24 h treatment with 10 nM–100 µM, an increase in the ROS level was observed (up to 16%, compared to the control), and a similar tendency was observed after 48 h of treatment with all Les-6166 doses (increasing from 8% to 20%, compared to the control) (Figure 3G–I). In turn, Les-6009 increased the ROS level in the A549 cells only at the 100 µM concentration after 6 h (19%, compared to the control). After the 24 h and 48 h treatments, no notable changes in ROS production were observed (Figure 3G–I). The SH-SY5Y cells treated with Les-6009 in the concentration range of 100 nM–100 µM for 6 h showed a decline in ROS production (up to 15%, compared to the control). Interestingly, after the 24 h and 48 h exposure to all doses of Les-6009, a decrease in ROS production was noticed (from 15% to 45%, compared to the control) (Figure 3G–I). After the 6 h treatment of the CACO-2 cells with Les-6009 in the range of 10 nM–100 µM, a decrease in ROS production was observed, compared to the control (up to 31%). A similar tendency was observed after the 24 h treatment of the CACO-2 cells, i.e., a decrease in ROS production up to 28%, compared to the control. However, after the 48 h treatment, the CACO-2 cell line was characterized by an increasing ROS level at the 50 and 100 µM concentrations (20% and 31%, respectively, compared to the control) (Figure 3J–L).

After the 6 h treatment with Les-6166, the BJ cells exhibited increased ROS production at the 10, 50, and 100 µM concentrations (up to 12%, 10%, and 9%, respectively, compared to the control). After 24 h of exposure, an increase in ROS production was detected in the concentration range from 50 nM to 100 µM, analogically to the 48 h treatment results, i.e., an increase in ROS production from 15% to 35%, compared to the control (for the 50 nM–100 µM concentrations) (Figure 3J–L). After the 6 h treatment with the tested compound at the 1 nM–1 µM concentrations, the A549 cells showed increased ROS production (up to 29%, compared to the control), similarly to the 24 h treatment with Les-6009 (with an increase of up to 23%, compared to the control). However, after the 48 h treatment, no significant changes in ROS production were observed (Figure 3J–L). The SH-SY5Y cells did not show any changes in ROS production after the 6 h treatment with Les-6166. After the 24 h and 48 h treatments, Les-6166 caused an increase in ROS production at all concentrations (from 10% to 27%, compared to the control) (Figure 3J–L).

### 2.3. Impact of 4-Thiazolidinone Derivatives on the Caspase-3 Activity

**Figure 4:**
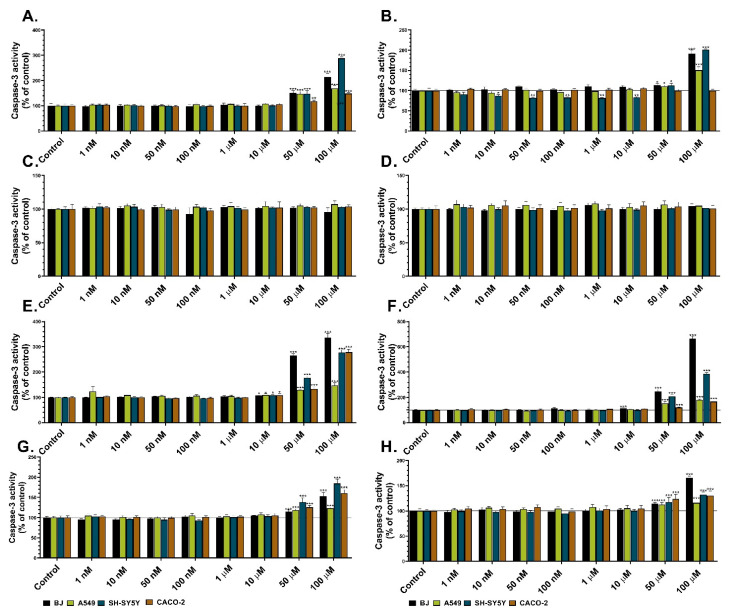
Caspase-3 activity in BJ, A549, SH-SY5Y, and CACO-2 cell lines after 24 h (**A**,**C**,**E**,**G**) and 48 h treatments (**B**,**D**,**F**,**H**) with Les-3166 (**A**,**B**), Les-5935 (**C**,**D**), Les-6009 (**E**,**F**), and Les-6166 (**G**,**H**). Mean values (*n* = 6) with standard deviation (error bars) where *, **, and *** are statistically different from the respective (vehicle-treated) control at *p* < 0.05, *p* < 0.01, and *p* < 0.001, respectively (one-way ANOVA, Tukey’s test).

Caspase-3 activity is a well-known indicator of apoptosis induction in cells. Therefore, this assay was used to determine the impact of the studied heterocycles on apoptosis induction in normal (BJ) and three cancer cell lines (A549, SH-SY5Y, and CACO-2). Les-3166 caused a considerable increase in caspase-3 activity in all cell lines at the 50 and 100 µM concentrations after the 24 h and 48 h treatments (from 50% to 188 %, compared to the control) (Figure 4C,D). Moreover, after the 48 h Les-3166 treatment, caspase-3 activity was increased also in the BJ cells (up to 13%, compared to the control) at the 50 nM, 1 µM, and 10 µM concentrations (Figure 4C,D). In turn, Les-5935 did not influence caspase-3 activity in comparison to the control in any cell line or concentration of the compound after the 24 h and 48 h treatments (Figure 4A,B). After the 24 h exposure to 10–100 µM of Les-6009, caspase-3 activity increased substantially in the BJ, A549, and CACO-2 cells (from 9% to 237%, compared to the control). A similarly high increase was noted in the SH-SY5Y cells (up to 177%, compared to the control) exposed to 50–100 µM of the compound (Figure 4G,H). After the 48 h treatment, an increase in caspase-3 activity was observed in all studied cell lines treated with the 50 and 100 µM concentrations (up to 295%, compared to the control). After the 24 h and 48 h Les-6166 exposure, caspase-3 activity increased only at the 50 and 100 µM concentrations in all studied cell lines (up to 84%, compared to the control) (Figure 4E,F). Similar effects were observed after the 48 h cell treatment with Les-6166 (from 13% to 65%).

### 2.4. Impact of 4-Thiazolidinone Derivatives on the Cell Cycle

**Figure 5:**
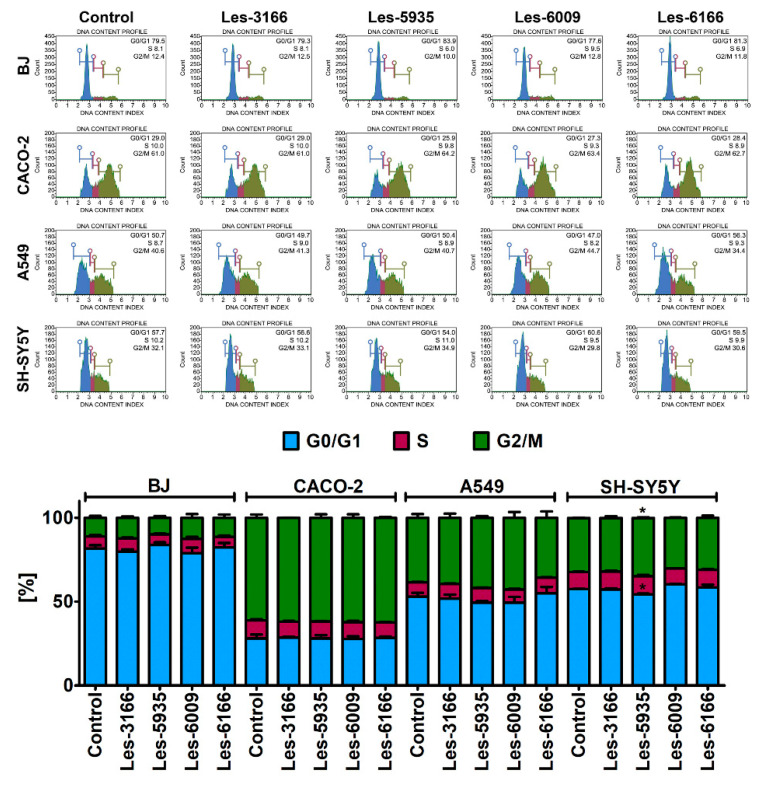
Cell cycle analysis after Les-3166, Les-5935, Les-6009, and Les-6166 treatments for 24 h in BJ, A549, SH-SY5Y, and CACO-2 cells. Mean values with standard deviation (error bars) where * are statistically different from the respective (vehicle-treated) control at *p* < 0.05 (one-way ANOVA, Tukey’s test) (**bottom**). Representative histograms are also shown (**top**).

Flow cytometry analysis was used to determine whether the presented 4-thiazolidinone derivatives exerted a cytostatic effect on three cancer cell lines (A549, SH-SY5Y, and CACO-2). The 10 µM concentration of each compound, indicated by the results of the resazurin reduction (Figure 2) and caspase-3 activity assays, was chosen for the cell cycle analysis (Figure 4). None of the compounds (Les-5935, Les-3166, Les-6166, and Les-6009) induced notable changes in the G0/G1, S, or G2/M phases of the cell cycle (Figure 5). However, some minor but significant changes were observed for Les-5935 only after the 24 h exposure in the SH-SY5Y cells (Figure 5). In the Les-5935-treated SH-SY5Y cells, an increase in the G2/M cell subpopulation was revealed, whereas the Les-5935 stimulation reduced the levels of cells at the G0/G1 phase of the cell cycle (Figure 5).

### 2.5. Impact of 4-Thiazolidinone Derivatives on Biofilm Eradication

*Pseudomonas aeruginosa*, which is responsible inter alia for antibiotic resistance in hospital treatment, was chosen to evaluate the bacterial biofilm eradication activity of the tested 4-TZDs. The biofilm degradation level was determined by crystal violet staining.

**Figure 6:**
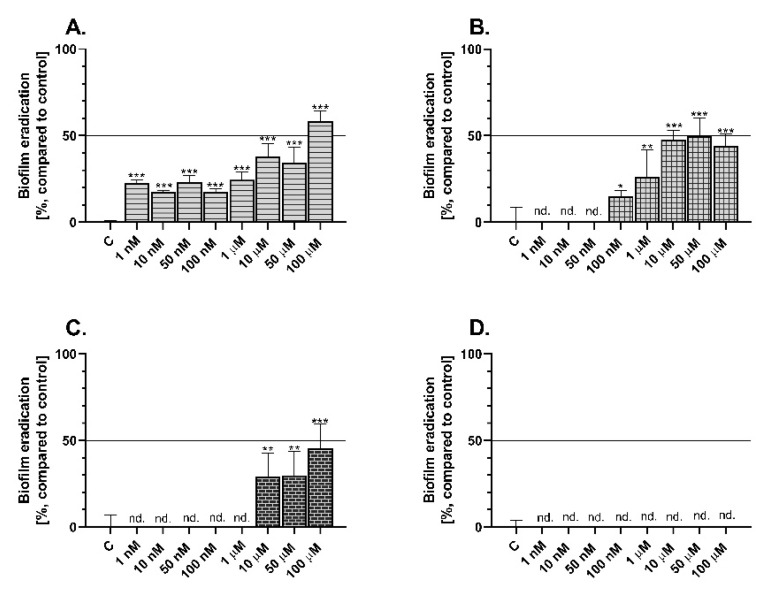
*Pseudomonas aeruginosa*-based biofilm eradication after 24 h treatment with Les-3166 (**A**), Les-5935 (**B**), Les-6009 (**C**), and Les-6166 (**D**). Mean values (*n* = 6) with standard deviation (error bars) where *, **, and *** are statistically different from the respective (vehicle-treated) control at *p* < 0.05, *p* < 0.01, and *p* < 0.001, respectively (one-way ANOVA, Tukey’s test); nd—no changes in biofilm eradication were detected, in comparison to the control denoted as “C”.

Les-3166 caused significant degradation of the *P. aeruginosa* biofilm after 24 h exposure to all tested concentrations. However, the highest antibacterial effect was observed in the treatments with 10, 50, and 100 µM, i.e., 37%, 34%, and 58%, respectively, compared to the control (Figure 6A). In turn, Les-5935 increased bacterial biofilm eradication only in the concentration range from 100 nM to 100 µM, up to 49%, compared to the control (Figure 6B). Les-6009 showed an ability to inhibit *P. aeruginosa*-based biofilm formation at the concentrations of 10, 50, and 100 µM, causing a 28%, 29%, and 45% decrease in biofilm biomass, respectively, compared to the control (Figure 6C). Interestingly, Les-6166 did not cause any changes in biofilm eradication at any tested concentration (Figure 6D).

## 3. Discussion

Despite the great progress in cancer treatment and the development of novel therapeutic strategies, cancer morbidity is still increasing. Some research reports that nearly 16% of people suffering from some cancer types have a low chance of survival [37,38]. Many drugs commonly used in chemotherapy are characterized by sophisticated structures based on heterocyclic cores [2,39]. Unfortunately, multidrug resistance has been observed in a variety of cancer cells nowadays [40,41]. Therefore, searching for new biologically active compounds bearing heterocyclic core(s) may be one of the fruitful directions in the development of novel anticancer agents. One of the classes of heterocycles with promising anticancer activity is the series of 4-thiazolidinone (4-TZD) derivatives. To date, it has been described that various 4-thiazolidinones exhibit anticancer properties demonstrated in numerous in vitro and in vivo models [10,12,13,14,18,25,26,27,28]. The determination of the impact of xenobiotics on metabolic activity is one of the most relevant analyses allowing the formulation of preliminary assumptions about their application potential. In this study, we chose this method to perform in vitro screening of four 4-thiazolidinone derivatives, i.e., the potential impact of Les-5935, Les-3166, Les-6166, and Les-6009 on the metabolism of BJ, A549, SH-SY5Y, and CACO-2 cells. There are a few reports proving that, e.g., Les-5935 and Les-6166 have activity towards certain cytochrome P450 isoforms in healthy mouse fibroblasts (3T3-L1 cell line) [28]. Therefore, the impact of the tested 4-thiazolidinone derivatives on the metabolic activity of normal human fibroblasts was evaluated. The results have shown that, generally, Les-5935 does not affect negatively the BJ cell line, in contrast to Les-3166, Les-6166, and Les-6009, which are able to significantly decrease the metabolic activity in BJ. Similar results were obtained by Szychowski et al., who proved that Les-5935, even after 72 h treatment, did not show cytotoxicity against mouse fibroblasts [28]. However, Les-6166 decreased the metabolic activity in the 3T3-L1 mouse cell line after 48 h treatment in a concentration range of 10–100 µM [28]. The authors also proved that compounds Les-5935 and Les-6166 affected the expression and/or activity of cytochrome P450, especially the Cyp1b1 form. In the present study, all tested compounds decreased the metabolic activity of the A549 cell line. However, the most desirable effects were observed for Les-5935 and Les-6166, which did not influence the metabolic activity of the healthy fibroblasts [28]. However, these 4-thiazolidinone derivatives have never been tested as potential agents for lung cancer therapy. This is particularly important due to the high death rate in this cancer type. Moreover, many lung carcinoma cells (up to 80% in non-small cell lung carcinoma) are characterized by resistance to traditional chemotherapy [42]. Our data show that Les-6009 had a potent inhibitory activity against A549 cells (up to 50% inhibition of metabolic activity, compared to untreated cells), which was not observed for Les-3166 and Les-6166. Similar results were obtained by El Sayed et al., who proved that one of the tested nitro-heterocycles was able to significantly decrease A549 metabolic activity in comparison to the control group [43]. Moreover, the authors proved that this heterocycle showed lower IC_50_ than doxorubicin, which is considered to be one of the most common drugs used in lung cancer therapy [43]. Beloglazkina et al. have reported that N-unsubstituted heterocyclic moieties are able to significantly affect the metabolic activity in the lung carcinoma cell line [44]. We showed that high Les-5935 and Les-6009-induced toxicity was not accompanied by ROS production after 24 h or 48 h treatment. Therefore, we suppose that the observed cytotoxic effect was ROS-independent. This outcome is surprising. Many reports have highlighted the correlation between the presence of heterocycles and an increase in ROS production. For instance, Arambula et al. proved upregulation of endoplasmic reticulum stress response pathway-related genes, resulting in, e.g., higher toxicity [45]. However, we suppose that the significant toxic effects of these two compounds (Les-5935 and Les-6009) toward the lung cancer cell line are related to their high bioavailability. Similar outcomes were presented by Zhang et al., who confirmed high concentrations of studied heterocycles in cancer cells just after 4 h of treatment [46]. Additionally, our results have shown that only Les-3166 decreases metabolic activity in the CACO-2 cell line. Similar results were obtained for related thiazolidinones and the ability to induce apoptosis was shown [18,25,26,27,28,34,35,47]. Moreover, colon cancer is considered to be one of the most difficult cancer types for treatment due to its high drug resistance and production of large amounts of extracellular matrix [48]. Additionally, Asif et al. showed that N-heterocyclic carbenes similarly decreased the metabolic activity in the CACO-2 cell line [49]. Therefore, it is worth highlighting that Les-6009 showed potent inhibitory activity against the SH-SY5Y cell line, even when used at the nanomolar concentrations. A high neurodegenerative effect of pyrazolopyridine-based heterocycles in SH-SY5Y cells and an increase in apoptosis-related protein in the cells were shown by Jouha et al. [50]. Summarizing, our results show that the most promising effect on metabolic activity occurs in the A549 cells, which were characterized by a dose-dependent decrease in this parameter in the concentration range between 10 nM–100 µM, which may give a strong basis to the further determination of the mechanism of action of these compounds, especially in the A549 cells. Interestingly, in the SH-SY5Y and CACO-2 cells the most promising effect was noticed in the micromolar concentrations, therefore the using of these compounds in such cells is limited. ROS-dependent toxicity is the main mechanism engaged in toxicity effects caused by drugs in many cancer and normal cells. Many anticancer drugs are able to increase intracellular ROS generation and subsequently cause cell damage [51,52]. Similar mechanisms were found to occur in cells after 4-TZD uptake, causing oxidative stress in vitro in different cell types, e.g., SCC-15, CACO-2, or A549 [27,34]. Moreover, Szychowski et al. showed that 4-TZD can act via the PPARγ-dependent pathway [25]. Therefore, to extend the knowledge of the potential mechanisms of action of the studied 4-thazolidinones, ROS production analysis was performed using the highly sensitive H_2_DCFDA probe. Our results have demonstrated that Les-3166 does not cause a significant increase in ROS production in BJ cells. However, the other compounds (Les-5925, Les-6009, and Les-6166) increased ROS levels in the A549 cell line, in comparison to the control. This phenomenon is similar to that reported by Armentano et al., who proved that oxazolidinone heterocycle derivatives were able to influence the mitochondrial function and subsequently increase ROS generation and induce apoptosis [53]. Similar results were presented by Szychowski et al. in their comprehensive studies on the impact of 4-TZD on apoptosis induction in many cell lines, e.g., SCC-15, A549, 3T3-L1, or SH-SY5Y [25,27,28]. Les-6166 and Les-6009 notably decreased ROS production in the CACO-2 and SH-SY5Y cells, respectively. This may prove their antioxidant properties and confirm their role as antiradical agents/free radical scavengers [54]. As shown by Szychowski et al., some of the tested heterocycles are characterized by antioxidant properties and potential to decrease oxidative stress induced by H_2_O_2_ treatment [27]. Moreover, Lincoln et al. showed that N-heterocyclic amine chelates scavenged ROS in primary human fibroblasts [55]. As suggested by the presented data, the potential of 4-thazolidinones to induce/decrease ROS production varies and depends on the cell type and concentrations used. Moreover, both effects can be useful in the design of new drug-like molecules [56,57], as many studies have shown a correlation between ROS overproduction in cells and apoptosis induction. Therefore, in the subsequent stage of our study, we have decided to evaluate whether the tested heterocycles were able to induce apoptosis.

Apoptosis is one of the most desirable methods to remove cancer cells from organisms via safe cell death processes that decrease inflammation [58]. There are many groups of proteins, which are characteristic for induced apoptosis. The most common marker of apoptosis is the increase in the activity of caspase-3, representing effector proteins. Therefore, we chose caspase-3 activity to determine the impact of Les-5935, Les-3166, Les-6166, and Les-6009 on the apoptosis induction ability. Our results showed that Les-5935 did not induce apoptosis in any tested cell lines. This outcome is similar to the effect on the 3T3-L1 cell line shown by Szychowski et al., proving that this heterocycle cannot induce apoptosis in normal and cancer cells [28]**.** However, the other compounds showed the ability to induce apoptosis in the BJ, A549, SH-SY5Y, and CACO-2 cell lines, but only at two of the tested concentrations (50 and 100 µM). Senkiv et al. studied the mechanism of apoptosis induction by (*Z)*-5-[5-(2-hydroxyphenyl)-3-phenyl-4,5-dihydropyrazol-1-ylmethylene]-3-(3-acetoxyphenyl)-2-thioxothiazolidin-4-one and proved that this compound was able to significantly increase apoptosis induction. Moreover, this effect was comparable with that of doxorubicin [34]. In turn, Vial et al. concluded that induction of apoptosis was strictly related to drug metabolism in the cell and potential pharmacokinetics in vivo [59]. However, we suppose that further investigations in this field need to be performed.

The metabolic activity results proved a toxicity effect of the tested heterocycles on the normal and cancer cell lines. Many chemotherapeutics show a cytostatic effect (cell growth inhibitory effect), which can be accompanied by cell cycle arrest [60]. 4-Thiazolidinone derivatives are considered to exert a cytostatic effect, as highlighted by Kulchitsky et al. [61] and Gidwani et al. [62]. Therefore, based on ROS production, caspase-3 activity, and resazurin reduction results, we chose the 10 µM concentration of each heterocycle compounds as an effective but nontoxic dose to evaluate the impact of Les-5935, Les-3166, Les-6166, and Les-6009 on cell cycle progression. Our results showed that none of the tested compounds possessed cytostatic effects, as indicated by the unaffected subpopulations at the G0/G1, S, or G2/M phases of the cell cycle, compared to the control. Opposite results were obtained by Senkiv et al., who revealed that 4-thiazolidinone treatment in HL-60 cells resulted in G0/G1 cell cycle arrest [34]. Hence, due to the variety and differences in their chemical features, it is rather difficult to predict the cytostatic or cytotoxic properties of small 4-thiazolidinone-based compounds and related heterocycles. Based on present results, it can be concluded that all of the tested 4-thiazolidinones exert a cytotoxic rather than cytostatic effect. It is worth emphasizing that mainly micromolar rather than nanomolar ranges are the effective concentrations of Les-5935, Les-3166, Les-6166, and Les-6009.

Many heterocycles are used in the search for new compounds that may be potentially more effective to antibiotic-resistant bacterial strains. This is very important especially in the case of strains which are able to form biofilms with a structure preventing contact of the bacterial surface with the xenobiotic. Given scientific reports indicating that 4-thiazolidinones can be characterized by some antibacterial potential [12,29], we decided to determine whether Les-3166, Les-5935, Les-6009, and Les-6166 can cause degradation of bacterial biofilm formed by *P. aeruginosa*. Our results have demonstrated that Les-3166 possesses high antibacterial properties, as it caused a decrease in biofilm biomass at the concentration range from 1 nM to 100 µM. However, it caused 50% eradication of the bacterial biofilm only the concentration of 100 µM. Additionally, Les-5935 and Les-6009 exert less potent antibacterial effects, especially Les-6009, which reduced biofilm biomass only in the concentration range from 10 to 100 µM. Vikrant et al. highlighted that some synthesized 4-TZD showed high bactericidal activity, causing inhibition of *Escherichia coli*, *Klebsiella pneumoniae*, *Bacillus cereus*, and *Salmonella typhi* growth; moreover, these effects were stronger than those of the sulphametoxazol or ciprofloxacin drugs [63]. As reported by Omari et al., 10 newly synthesized 4-TZD showed high antifungal and antibacterial activities, also against *P. aeruginosa* [64]. Considering the literature data cited above and our results, it can be concluded that Les-3166, Les-5935, and Les-6009 can be valuable compounds in the treatment of antibiotic-resistant bacterial strains. This finding is important for the design of molecules with dual anticancer and antimicrobial action [65,66]. In turn, Les-6166 does not show any effect on biofilm degradation, which clearly proves that each 4-TZD compound needs to be tested for its own properties.

## 4. Materials and Methods

### 4.1. Reagents

Trypsin, penicillin, streptomycin, dimethyl sulfoxide (DMSO), caspase-3 substrate (Ac-DEVD-pNA), 2′,7′-dichlorodihydrofluorescein diacetate (H2DCF-DA), hydrogen peroxide (H_2_O_2_), resazurin, hydroxyethyl piperazine ethanesulfonic acid (HEPES), sodium chloride, 3-[(3-cholamidopropyl)dimethylamino]-1-propanesulfonate hydrate (CHAPS), ethylenediaminetetraacetic acid (EDTA), glycerol, dithiothreitol (DTT), ethanol, acetic acid, and crystal violet (CV) were purchased from Sigma-Aldrich (St. Louis, MO, USA). F12-K, DMEM, DMEM/F12, MEM, and phosphate-buffered saline without Ca^2+^ and Mg^2+^ were purchased from Corning (Tewksbury, MA, USA). Fetal bovine serum (FBS) and fetal bovine serum heat inactivated (FBS HI) were purchased from EURx (Gdańsk, Poland). The LB medium was purchased from BTL (Lodz, Poland). All stock solutions used in this work were prepared by dissolving the compound powder in DMSO to reach a 100 mM solution. The obtained concentrations were further used to yield 1, 10, 50, and 100 nM and 1, 10, 50, and 100 µM solutions using DMSO as a solvent.

### 4.2. Synthesis of Heterocyclic Compounds

4-Thiazolidinones Les-3166 [18], Les-6009 [67] Les-6166 [28], and thiopyranothiazole derivative Les-5935 [28] were synthesised according to previously developed methods. The chemical structures of 4-TZD are presented in Figure 1.

### 4.3. Cell Lines and Microorganism Cultures

Human foreskin fibroblasts BJ (ATCC CRL-2522), human epithelial lung carcinoma A549 (ATCC CCL-185), human neuroblastoma SH-SY5Y (ATCC CRL-2266), and colorectal adenocarcinoma CACO-2 were obtained from the American Type Culture Collection (ATCC, distributor: LGS Standard, Łomianki, Poland). The BJ and CACO-2 cells were cultured in MEM medium supplemented with 10% FBS and 2 mM L-glutamine. The A549 cells were cultured in F12-K medium supplemented with 10% FBS. The SH-SY5Y cells were maintained in DMEM/F12 medium supplemented with 10% FBS HI. All media were supplemented with 1% *v*/*v* penicillin and streptomycin. The cells were cultured in a humidified atmosphere with 5% CO_2_ at 37 °C until they reached confluence. Next, the cells were seeded in 96-well plates at a density of 5 × 10^3^ per well (for 6 h, 24 h, and 48 h treatments) and precultured for 24 h before the experiment. Subsequently, the medium was removed and fresh ones with rising concentrations of Les-3166, Les-5935, Les-6009, and Les-6166, namely 1, 10, 50, and 100 nM and 1, 10, 50, and 100 µM, were added. The final concentration of DMSO in the medium was always 0.1%. In this study, cells treated with DMSO (vehicle) were always used as a control.

*Pseudomonas aeruginosa (*ATCC 27853) obtained from the University of Information Technology and Management collection were subcultured overnight in LB medium with shaking (150 rpm) at 30 °C in a flat-bottom flask. Afterwards, OD was measured, and the inoculum was diluted in a 1:100 ratio (bacteria culture: fresh medium). Next, the bacteria were subjected to crystal violet staining, in which the antibacterial potential of the tested heterocycles was determined.

### 4.4. Cell Viability Assay

The resazurin reduction viability assay was used to determine the effect of the studied compounds on cell metabolic activity. Resazurin is a nonfluorescent nontoxic dye which penetrates living cells and is subsequently converted to fluorescent resorufin by mitochondrial enzymes. The protocol was performed according to Czekanska [68] with minor modifications. Briefly, a working solution (600 µM) of resazurin was prepared and stored at 4 °C. After 6 h, 24 h, and 48 h treatments with the compounds, the medium was removed and replaced with a fresh one containing the resazurin working solution and 1% FBS. Subsequently, the culture was kept at 37 °C and 5% CO_2_ for 30 min. After this time, the fluorescence was measured at 530 nm excitation and 590 nm emission wavelengths on a microplate reader (FiletrMax F5).

### 4.5. ROS Production

The intracellular ROS production was measured by application of the 2′,7′-dichlorofluorescein (H_2_DCF-DA) dye after 6 h, 24 h, and 48 h treatments with Les-3166, Les-5935, Les-6009, and Les-6166. This fluorescent probe is able to penetrate live cells and is deacetylated by esterases into a nonfluorescent substance which is further oxidized to fluorescent H_2_DCF-DA by generated ROS. The ROS production assay was performed according to Szychowski et al. [27]. Briefly, the BJ, A549, SH-SY5Y, or CACO-2 cells were incubated with the 5 µM working solutions of H_2_DCF-DA in serum-free and phenol red-free medium for 40 min before the treatment with the compounds to evaluate the level of ROS production. After this time, the medium was removed, the cells were washed once with PBS, and a fresh medium with rising concentrations of the heterocycles was added. After 6 h, 24 h, and 48 h incubation, fluorescence of H_2_DCF-DA was measured using a microplate reader (FilterMax F5) at maximum excitation (485 nm) and emission (535 nm) spectra. A 0.03% H_2_O_2_-solution was used as a positive control (data not shown).

### 4.6. Caspase-3 Activity

Caspase-3 is a well-known indicator of apoptosis induction. Therefore, in this study, caspase-3 activity analysis was chosen to evaluate the impact of the tested heterocycles on apoptosis induction. The assay was performed according to Szychowski et al. [26]. Briefly, cultured cells were disintegrated after 24 h and 48 h by adding 50 µL of lysis buffer per well (50 mM HEPES, pH 7.4, 100 mM NaCl, 0.1% CHAPS, 1 mM EDTA, 10% glycerol, and 10 mM DTT) and kept for 10 min at 4 °C. Subsequently, the caspase-3 substrate (Ac-DEVD-pNA) was added to the lysates and incubated for 30 min at 37 °C. After this time, the absorbance (405 nm wavelength) of the mixture was measured using a microplate reader (FilterMax F5).

### 4.7. Flow Cytometry-Based Cell Cycle Analysis

Flow cytometry was used to evaluate 4-TZD derivative-mediated changes in the cell cycle in the normal (BJ) and cancer (A549, SH-SY5Y, and CACO-2) cell lines. The analysis was performed according to Lewińska et al. [69] using a Muse™ Cell Analyzer and a Muse™ Cell Cycle Kit. Briefly, BJ, A549, SH-SY5Y, and CACO-2 were seeded in 25-cm^2^ culture flasks at a density of 1 × 10^6^ cells in MEM with 10% FBS, F-12K with 10% FBS, DMEM/F12 with 10% FBS HI, and MEM with 10% FBS, respectively. After 24 h, the medium was removed and replaced with a fresh one containing serum and a 10 µM concentration of Les-3166, Les-5935, Les-6009, and Les-6166. After 24 h treatment, the medium was removed. The cells were washed 3 times with PBS and detached by a trypsin solution. After centrifugation (10 min, 200× *g*), the supernatant was removed and the cell pellet was resuspended in 70% EtOH and frozen at −20 °C until the experiment. Shortly before the procedure, the cell samples were washed with DPBS, and the percentage of cells in the G0/G1, S, and G2/M phase was assessed according to the producer’s protocol (Luminex Corporation, Austin, TX, USA).

### 4.8. Antibacterial Potential of 4-Thiazolidinone Derivatives

*Pseudomonas aeruginosa* was chosen to evaluate the antibacterial effect of Les-3166, Les-5935, Les-6009, and Les-6166. This bacterial strain is able to form an antibiotic-resistant biofilm, which is considered to be an emerging problem in modern hospital infections. The crystal violet staining assay was performed according to O’Toole with minor modifications [70]. Briefly, *P. aeruginosa* was cultured overnight in LB medium at 30 °C with shaking (150 rpm). Afterwards, the inoculum was diluted in a 1:100 ratio (bacteria: fresh medium, respectively), seeded in a sterile 96-well plate (100 µL/well), and cultured. After 24 h, a biofilm was formed at the bottom of the wells. Subsequently, the medium was removed and replaced gently with a fresh one containing increasing concentrations of Les-3166, Les-5925, Les-6166, and Les-6009. After 24 h treatment, the medium was removed, and the biofilm was washed once with PBS. Next, a 0.1% CV solution was added to each well and stained for 15 min. After this time, each well was washed 5 times with distilled water to remove excess dye. Afterwards, the biofilms were dried at 40 °C for 3 h. Subsequently, CV was solubilized with a 0.33% acetic acid solution, transferred to a new plate, and measured in a microplate reader (FilterMax F5) at a 550 nm wavelength.

### 4.9. Statistical Analysis

Data are presented as mean values ± standard deviations (SD). Each concentration treatment was repeated at least six times (*n* = 6); moreover, each experiment was repeated at least three times. Subsequently, these values were used to perform statistical analysis. In each result group (time intervals), one-way ANOVA with post-hoc Tukey’s test was performed using GraphPad Prism 8.0 Statistical Analysis Panel. The results were analyzed and always expressed as a percentage of the control (vehicle-treated cells). *—*p* < 0.05; **—*p* < 0.01, and ***—*p* < 0.001 in comparison to the control (vehicle-treated group).

## 5. Conclusions

Our study is the first to present wide in vitro screening of four 4-TZD derivatives for determination of their cytotoxic, cytostatic, and antibacterial effects. The results have shown that Les-3166 (2-{2-[3-(benzothiazol-2-ylamino)-4-oxo-2-thioxothiazolidin-5-ylidenemethyl]-4-chlorophenoxy}-N-(4-methoxyphenyl)-acetamide) is cytotoxic towards all cell lines tested at micromolar concentrations and mainly via the ROS-independent pathway. In turn, Les-5935 (7-oxa-10-thia-8-aza-cyclopenta[b]phenanthren-9-one) can induce high ROS generation in BJ and A549 cells, but its cytotoxicity is limited to these two cell lines. Les-6009 (5-fluoro-3-(4-oxo-2-thioxothiazolidin-5-ylidenemethyl)-1H-indole-2-carboxylic acid methyl ester) can affect significantly the A549, BJ, and SH-SY5Y cell lines by decreasing their metabolic activity, without causing oxidative stress in these cell lines. Les-6166 (5-fluoro-3-[2-(4-hydroxyphenylamino)-4-oxo-4H-thiazol-5-ylidenemethyl]-1H-indole-2-carboxylic acid methyl ester) shows the strongest prooxidative effect on the cell lines (BJ, A549, and SH-SY5Y); its cytotoxicity is time dependent (higher after 24 h treatment than 48 h treatment). The tested compounds do not show any significant cytostatic effects. However, Les-3166, Les-5935, and Les-6009 are able to eradicate *P. aeruginosa* bacterial biofilm, especially in the concentration range from 10 to 100 µM. Nevertheless, more research is needed to elucidate the precise mechanism of action of the studied compounds. Tested compounds are promising active substances in non-small cell lung cancer treatment. However, more research needs to be developed to fully elucidate the exact mechanism of these compounds in such cells. The usage of the new tested 4-TZD in the SH-SY5Y and CACO-2 cells is limited due to an observed toxic effect only in the micromolar concentrations.

## Data Availability

The datasets generated during and/or analyzed during the current study are available from the corresponding author on reasonable request.

## Supplementary Materials

The following supporting information can be downloaded. Table S1: Les-3166 6 h-treatment; Table S2: Les-3166 24 h-treatment; Table S3: Les-3166 48 h-treatment; Table S4: Les-5935 6 h-treatment; Table S5: Les-5935 24 h-treatment; Table S6: Les-5935 48 h-tratment; Table S7: Les-6009 6 h-treatment; Table S8: Les-6009 24 h-treatment; Table S9: Les-6009 48 h-treatment; Table S10: Les-6166 6 h-treatment; Table S11: Les-6166 24 h-treatment; Table S12: Les-6166 48 h-treatment.

## Sample Availability

Samples of the compounds Les-3166, Les-5935, Les-6009 and Les-6166 are available from the authors.
