# Supplementary material for: Evaluation of Anticancer and Antibacterial Activity of Four 4-Thiazolidinone-Based Derivatives
Source: Molecules. 2022 Jan 28;27(3):894. doi: 10.3390/molecules27030894 (PMC8839971; doi:10.3390/molecules27030894)
Supplement: Supplementary file 1 [file molecules-27-00894-s001.zip › molecules-1566308-supplementary.pdf]

Supplementary Data

Evaluation of anticancer and antibacterial activity of four 4-thiazolidinone-based derivatives

Bartosz Skóra, MSc.[1], Anna Lewińska, Ph.D., D.Sc.[2], Anna Kryshchyshyn-Dylevych Ph.D.[3], Danylo Kaminskyy Ph.D.[3], Roman Lesyk Ph.D., D.Sc., Professor[1,3], Konrad A. Szychowski, Ph.D., D.Sc., Assoc. Prof.[1]

[1] Department of Lifestyle Disorders and Regenerative Medicine, Medical College, University of Information Technology and Management in Rzeszow, Sucharskiego 2, 35-225 Rzeszow, Poland

[2] Department of Biotechnology, Institute of Biology and Biotechnology, College of Natural Sciences, University of Rzeszow, Pigonia 1, 35-310 Rzeszow, Poland

[3] Department of Pharmaceutical, Organic and Bioorganic Chemistry, Danylo Halytsky Lviv National Medical University, Pekarska 69, Lviv 79010, Ukraine

(Family name is underlined)

Corresponding author:

Bartosz Skóra, MSc.

Department of Lifestyle Disorders and Regenerative Medicine, Medical College, University of Information Technology and Management in Rzeszow, Sucharskiego 2, 35-225 Rzeszow, Poland. mail: bskora@wsiz.edu.pl

ORCID numbers:

Bartosz Skóra: **0000-0001-7833-9985**

Anna Lewińska: **0000-0001-8055-1918**

Anna Kryshchyshyn-Dylevych: **0000-0002-8713-7020**

Danylo Kaminskyy: **0000-0001-6837-367X**

Roman Lesyk: **0000-0002-3322-0080**

Konrad A. Szychowski: **0000-0003-2207-1160**

Statistical analyses of ROS production after 4-TZD treatment

1. Les-3166

1.1.6h-treatment:

Table S1. Data with *, **, *** are statistically different from the respective (vehicle-treated) control at p<0.05, p<0.01, and p<0.001, respectively (one-way ANOVA, Tukey test), ns. – no statistical differences.

Cell line	Dose	Value ± SD [%]	Statistical significance	Cell line	Dose	Value ± SD [%]	Statistical significance
BJ	Control	100.00 ± 2,87	-	A549	Control	100.00 ± 2,98	-
	1 nM	113.38 ± 13,92	ns.		1 nM	119.91 ± 9,29	***
	10 nM	111.38 ± 14,73	ns.		10 nM	120.02 ± 4,26	***
	50 nM	101.39 ± 13,75	ns.		50 nM	122.83 ± 7,10	***
	100 nM	101,08 ± 9,01	ns.		100 nM	119.06 ± 7,56	***
	1 µM	109.06 ± 6,41	ns.		1 µM	115.68 ± 11,84	*
	10 µM	112.34 ± 6,89	ns.		10 µM	119.97 ± 5,37	***
	50 µM	83.41 ± 6,39	ns.		50 µM	111.85 ± 4,00	ns.
	100 µM	89.89 ± 4,99	ns.		100 µM	123.21 ± 2,62	***
**Cell line**	**Dose**	**Value ± SD [%]**	**Statistical significance**	**Cell line**	**Dose**	**Value ± SD [%]**	**Statistical significance**
SH-SY5Y	Control	100.00 ± 5.98	-	CACO-2	Control	100.00 ± 7.18	ns.
	1 nM	97.90 ± 3.48	ns.		1 nM	99.61 ± 6.78	ns.
	10 nM	101.27 ± 4.55	ns.		10 nM	106.95 ± 8.78	ns.
	50 nM	102.06 ± 2.83	ns.		50 nM	100.11 ± 10.62	ns.
	100 nM	100.37 ± 3.28	ns.		100 nM	83.80 ± 3.06	*
	1 µM	99.99 ± 5.51	ns.		1 µM	87.20 ± 6.57	ns.
	10 µM	101.69 ± 4.17	ns.		10 µM	82.86 ± 6.86	**
	50 µM	103.30 ± 1.76	ns.		50 µM	87.55 ± 3.07	ns.
	100 µM	102.40 ± 4.79	ns.		100 µM	80.70 ± 10.84	**

1.2. 24-h treatment

Table S2. Data with *, **, *** are statistically different from the respective (vehicle-treated) control at p<0.05, p<0.01, and p<0.001, respectively (one-way ANOVA, Tukey test), ns. – no statistical differences.

Cell line	Dose	Value ± SD [%]	Statistical significance	Cell line	Dose	Value ± SD [%]	Statistical significance
BJ	Control	100.00 ± 7.87	-	A549	Control	100.00 ± 4.98	-
	1 nM	105.86 ± 9.50	ns.		1 nM	101.34 ± 9.16	ns.
	10 nM	99.52 ± 8.63	ns.		10 nM	102.65 ± 4.29	ns.
	50 nM	90.90 ± 7.93	ns.		50 nM	100.15 ± 2.23	ns.
	100 nM	95.09 ± 6.08	ns.		100 nM	103.15 ± 5.35	ns.
	1 µM	96.187 ± 5.72	ns.		1 µM	100.92 ± 9.00	ns.
	10 µM	96.69 ± 2.92	ns.		10 µM	104.78 ± 2.78	ns.
	50 µM	86.54 ± 3.92	*		50 µM	103.09 ± 1.10	ns.
	100 µM	71.567 ± 6.09	***		100 µM	106.73 ± 1.70	ns.
**Cell line**	**Dose**	**Value ± SD [%]**	**Statistical significance**	**Cell line**	**Dose**	**Value ± SD [%]**	**Statistical significance**
SH-SY5Y	Control	100.00 ± 1.87	-	CACO-2	Control	100.00 ± 9.04	ns.
	1 nM	99.56 ± 1.02	ns.		1 nM	96.63 ± 10.04	ns.
	10 nM	100.31 ± 0.74	ns.		10 nM	103.07 ± 10.25	ns.
	50 nM	100.43 ± 0.36	ns.		50 nM	100.38 ± 9.83	*
	100 nM	99.13 ± 1.87	ns.		100 nM	80.37 ± 8.45	ns.
	1 µM	99.36 ± 1.66	ns.		1 µM	82.10 ± 7.43	***
	10 µM	101.16 ± 0.81	***		10 µM	71.91 ± 8.62	***
	50 µM	105.67 ± 0.26	***		50 µM	73.85 ± 8.43	**
	100 µM	107.66 ± 0.22	ns.		100 µM	74.53 ± 14.64	**

1.3. 48h-treatment

Table S3. Data with *, **, *** are statistically different from the respective (vehicle-treated) control at p<0.05, p<0.01, and p<0.001, respectively (one-way ANOVA, Tukey test), ns. – no statistical differences.

Cell line	Dose	Value ± SD [%]	Statistical significance	Cell line	Dose	Value ± SD [%]	Statistical significance
BJ	Control	100.00 ± 8.98	-	A549	Control	100.00 ± 2.98	-
	1 nM	92.64 ± 9.35	ns.		1 nM	101.61 ± 10.58	ns.
	10 nM	89.66 ± 5.85	ns.		10 nM	104.16 ± 4.23	ns.
	50 nM	82.14 ± 9.90	**		50 nM	102.20 ± 1.90	ns.
	100 nM	80.97 ± 7.09	***		100 nM	102.14 ± 8.26	ns.
	1 µM	87.17 ± 2.53	ns.		1 µM	103.99 ± 4.76	ns.
	10 µM	88.47 ± 3.84	ns.		10 µM	105.04 ± 4.00	ns.
	50 µM	84.11 ± 5.32	**		50 µM	104.50 ± 1.32	ns.
	100 µM	80.09 ± 5.023	***		100 µM	107.31 ± 2.45	ns.
**Cell line**	**Dose**	**Value ± SD [%]**	**Statistical significance**	**Cell line**	**Dose**	**Value ± SD [%]**	**Statistical significance**
SH-SY5Y	Control	100.00 ± 3.87	-	CACO-2	Control	100.00 ± 12.5	-
	1 nM	102.44 ± 12.72	ns.		1 nM	103.77 ± 9.81	ns.
	10 nM	99.47 ± 1.94	ns.		10 nM	102.16 ± 12.41	ns.
	50 nM	99.60 ± 5.21	ns.		50 nM	91.74 ± 7.30	ns.
	100 nM	100.52 ± 5.02	ns.		100 nM	76.75 ± 8.09	**
	1 µM	99.91 ± 3.51	ns.		1 µM	80.19 ± 8.53	*
	10 µM	103.00 ± 5.37	ns.		10 µM	62.98 ± 9.13	***
	50 µM	103.21 ± 5.07	ns.		50 µM	71.91 ± 6.65	***
	100 µM	102.35 ± 7.63	ns.		100 µM	72.50 ± 10.67	***

2. Les-5935

2.1. 6h-treatment

Table S4. Data with *, **, *** are statistically different from the respective (vehicle-treated) control at p<0.05, p<0.01, and p<0.001, respectively (one-way ANOVA, Tukey test), ns. – no statistical differences.

Cell line	Dose	Value ± SD [%]	Statistical significance	Cell line	Dose	Value ± SD [%]	Statistical significance
BJ	Control	100.00 ± 3.96	-	A549	Control	100.00 ± 2.98	-
	1 nM	83.66 ± 6.75	**		1 nM	116.10 ± 7.29	ns.
	10 nM	87.10 ± 2.93	*		10 nM	121.85 ± 4.62	**
	50 nM	79.97 ± 8.44	***		50 nM	119.91 ± 10.65	**
	100 nM	99.09 ± 7.08	ns.		100 nM	121.13 ± 11.03	**
	1 µM	94.68 ± 9.21	ns.		1 µM	115.58 ± 7.48	ns.
	10 µM	115.76 ± 5.11	**		10 µM	122.82 ± 5.84	**
	50 µM	113.54 ± 4.16	*		50 µM	134.91 ± 13.77	***
	100 µM	127.08 ± 5.97	***		100 µM	138.64 ± 9.12	***
**Cell line**	**Dose**	**Value ± SD [%]**	**Statistical significance**	**Cell line**	**Dose**	**Value ± SD [%]**	**Statistical significance**
SH-SY5Y	Control	100.00 ± 2.25	-	CACO-2	Control	100.00 ± 5.82	-
	1 nM	100.62 ± 2.51	ns.		1 nM	94.82 ± 5.94	ns.
	10 nM	102.49 ± 0.79	ns.		10 nM	91.44 ± 6.65	ns.
	50 nM	102.16 ± 1.92	ns.		50 nM	96.65 ± 9.47	ns.
	100 nM	103.20 ± 0.40	ns.		100 nM	94.20 ± 6.65	ns.
	1 µM	99.94 ± 3.76	ns.		1 µM	95.16 ± 3.38	ns.
	10 µM	100.37 ± 4.48	ns.		10 µM	97.53 ± 2.39	ns.
	50 µM	99.21 ± 6.14	ns.		50 µM	94.78 ± 0.66	ns.

	100 µM	99.99 ± 1.80	ns.		100 µM	89.73 ± 13.11	ns.

2.2. 24h-treatment

Table S5. Data with *, **, *** are statistically different from the respective (vehicle-treated) control at p<0.05, p<0.01, and p<0.001, respectively (one-way ANOVA, Tukey test), ns. – no statistical differences.

Cell line	Dose	Value ± SD [%]	Statistical significance	Cell line	Dose	Value ± SD [%]	Statistical significance
BJ	Control	100.00 ± 3.12	-	A549	Control	100.00 ± 2.56	-
	1 nM	76.70 ± 8.22	***		1 nM	115.75 ± 9.52	ns.
	10 nM	79.90 ± 2.76	***		10 nM	137.07 ± 13.70	***
	50 nM	81.67 ± 8.91	***		50 nM	137.49 ± 6.61	***
	100 nM	80.09 ± 4.09	***		100 nM	124.09 ± 13.35	**
	1 µM	84.67 ± 8.59	**		1 µM	124.56 ± 12.57	**
	10 µM	114.45 ± 4.48	**		10 µM	111.86 ±10.32	ns.
	50 µM	114.23 ± 8.92	*		50 µM	107.33 ± 10.78	ns.
	100 µM	115.76 ± 4.92	**		100 µM	100.72 ± 3.82	ns.
**Cell line**	**Dose**	**Value ± SD [%]**	**Statistical significance**	**Cell line**	**Dose**	**Value ± SD [%]**	**Statistical significance**
SH-SY5Y	Control	100.00 ± 7.25	-	CACO-2	Control	100.00 ±2.98	-
	1 nM	100.19 ± 0.80	ns.		1 nM	133.86 ± 5.66	***
	10 nM	100.90 ± 0.35	ns.		10 nM	124.10 ± 12.82	***
	50 nM	100.56 ± 0.56	ns.		50 nM	107.41 ± 12.90	ns.
	100 nM	101.23 ± 0.22	ns.		100 nM	118.50 ± 11.94	*
	1 µM	100.07 ± 1.46	ns.		1 µM	108.38 ± 7.15	ns.
	10 µM	100.08 ± 1.65	ns.		10 µM	85.52 ± 11.70	ns.
	50 µM	99.84 ± 2.28	ns.		50 µM	90.04 ± 2.77	ns.
	100 µM	100.40 ± 0.57	ns.		100 µM	128.13 ± 1.60	***

2.3. 48h-treatment

Table S6. Data with *, **, *** are statistically different from the respective (vehicle-treated) control at p<0.05, p<0.01, and p<0.001, respectively (one-way ANOVA, Tukey test), ns. – no statistical differences.

Cell line	Dose	Value ± SD [%]	Statistical significance	Cell line	Dose	Value ± SD [%]	Statistical significance
BJ	Control	100.00 ± 2.68	-	A549	Control	100.00 ± 3.61	-
	1 nM	109.58 ± 9.53	ns.		1 nM	94.83 ± 4.00	ns.
	10 nM	115.25 ± 6.28	**		10 nM	87.91 ± 15.18	ns.
	50 nM	111.10 ± 5.07	ns.		50 nM	91.36 ± 9.14	ns.
	100 nM	120.91 ± 7.09	***		100 nM	89.42 ± 11.40	ns.
	1 µM	121.46 ± 9.26	***		1 µM	96.52 ± 7.27	ns.
	10 µM	160.84 ± 6.40	***		10 µM	97.05 ± 4.34	ns.
	50 µM	147.56 ± 7.77	***		50 µM	101.86 ± 2.52	ns.
	100 µM	149.09 ± 1.23	***		100 µM	103.43 ± 1.67	ns.
**Cell line**	**Dose**	**Value ± SD [%]**	**Statistical significance**	**Cell line**	**Dose**	**Value ± SD [%]**	**Statistical significance**
SH-SY5Y	Control	100.00 ± 2.26	-	CACO-2	Control	100.00 ± 8.23	-
	1 nM	103.10 ± 15.46	ns.		1 nM	103.80 ± 6.90	ns.
	10 nM	87.02 ± 12.88	ns.		10 nM	123.82 ± 7.64	***
	50 nM	90.66 ± 8.54	ns.		50 nM	124.68 ± 9.97	***
	100 nM	69.03 ± 6.03	***		100 nM	140.18 ± 16.34	***
	1 µM	65.61 ± 1.83	***		1 µM	103.17 ± 11.64	ns.
	10 µM	67.07 ± 2.85	***		10 µM	105.33 ± 4.14	ns.
	50 µM	79.47 ± 10.44	**		50 µM	106.37 ± 1.09	ns.
	100 µM	72.09 ± 4.36	***		100 µM	114.00 ± 4.02	ns.

3. Les-6009

3.1.6h-treatment

Table S7. Data with *, **, *** are statistically different from the respective (vehicle-treated) control at p<0.05, p<0.01, and p<0.001, respectively (one-way ANOVA, Tukey test), ns. – no statistical differences.

Cell line	Dose	Value ± SD [%]	Statistical significance	Cell line	Dose	Value ± SD [%]	Statistical significance
BJ	Control	100.00 ± 4.41	-	A549	Control	100.00 ± 6.92	-
	1 nM	89.35 ± 8.29	ns.		1 nM	109.61 ± 11.67	ns.
	10 nM	92.70 ± 9.68	ns.		10 nM	103.94 ± 7.63	ns.
	50 nM	95.97 ± 8.54	ns.		50 nM	103.14 ± 5.04	ns.
	100 nM	99.37 ± 2.63	ns.		100 nM	105.36 ± 7.96	ns.
	1 µM	101.19 ± 7.41	ns.		1 µM	106.40 ± 13.77	ns.
	10 µM	91.16 ± 8.97	ns.		10 µM	105.30 ± 8.83	ns.
	50 µM	88.92 ± 5.04	ns.		50 µM	104.50 ± 3.14	ns.
	100 µM	80.32 ± 2.56	***		100 µM	119.83 ± 6.42	**
**Cell line**	**Dose**	**Value ± SD [%]**	**Statistical significance**	**Cell line**	**Dose**	**Value ± SD [%]**	**Statistical significance**
SH-SY5Y	Control	100.00 ± 6.24	-	CACO-2	Control	100.00 ± 2.36	-
	1 nM	99.23 ± 8.98	ns.		1 nM	100.47 ± 4.08	ns.
	10 nM	92.99 ± 6.91	ns.		10 nM	83.81 ± 3.78	**
	50 nM	100.78 ± 11.69	ns.		50 nM	82.93 ± 7.79	**
	100 nM	92.20 ± 6.53	ns.		100 nM	77.90 ± 9.24	***
	1 µM	85.06 ± 2.25	*		1 µM	73.992 ± 6.84	***
	10 µM	89.58 ± 6.43	ns.		10 µM	76.49 ± 8.49	***
	50 µM	86.48 ± 3.15	*		50 µM	69.23 ± 4.30	***
	100 µM	87.31 ± 5.97	ns.		100 µM	84.35 ± 9.43	**

3.2. 24h-treatment

Table S8. Data with *, **, *** are statistically different from the respective (vehicle-treated) control at p<0.05, p<0.01, and p<0.001, respectively (one-way ANOVA, Tukey test), ns. – no statistical differences.

Cell line	Dose	Value ± SD [%]	Statistical significance	Cell line	Dose	Value ± SD [%]	Statistical significance
BJ	Control	100.00 ± 5.76	-	A549	Control	100.00 ± 9.29	-
	1 nM	103.75 ± 7.59	ns.		1 nM	99.84 ± 17.67	ns.
	10 nM	109.56 ± 6.83	ns.		10 nM	90.95 ± 18.56	ns.
	50 nM	105.19 ± 5.45	ns.		50 nM	95.09 ± 5.55	ns.
	100 nM	108.36 ± 2.37	ns.		100 nM	97.19 ± 11.77	ns.
	1 µM	106.10 ± 3.68	ns.		1 µM	101.33 ± 8.88	ns.
	10 µM	116.50 ± 5.76	***		10 µM	103.97 ± 14.30	ns.
	50 µM	109.96 ± 9.15	ns.		50 µM	102.51 ± 0.97	ns.
	100 µM	110.62 ± 2.64	ns.		100 µM	104.44 ± 13.11	ns.
**Cell line**	**Dose**	**Value ± SD [%]**	**Statistical significance**	**Cell line**	**Dose**	**Value ± SD [%]**	**Statistical significance**
SH-SY5Y	Control	100.00 ± 16.78	-	CACO-2	Control	100.00 ± 3.09	-
	1 nM	92.79 ± 14.41	ns.		1 nM	107.74 ± 2.50	ns.
	10 nM	85.63 ± 8.62	ns.		10 nM	85.83 ± 10.13	*
	50 nM	85.24 ± 7.86	ns.		50 nM	89.21 ± 8.89	ns.
	100 nM	83.65 ± 9.53	ns.		100 nM	81.78 ± 6.81	**
	1 µM	69.38 ± 3.04	***		1 µM	77.66 ± 8.42	***
	10 µM	72.11 ± 8.35	***		10 µM	76.88 ± 8.38	***
	50 µM	71.99 ± 0.47	***		50 µM	68.13 ± 3.46	***
	100 µM	81.18 ± 5.81	*		100 µM	85.28 ± 6.60	*

3.3. 48h-treatment

Table S9. Data with *, **, *** are statistically different from the respective (vehicle-treated) control at p<0.05, p<0.01, and p<0.001, respectively (one-way ANOVA, Tukey test), ns. – no statistical differences.

Cell line	Dose	Value ± SD [%]	Statistical significance	Cell line	Dose	Value ± SD [%]	Statistical significance
BJ	Control	100.00 ± 6.99	-	A549	Control	100.00 ± 4.10	-
	1 nM	107.40 ± 9.50	ns.		1 nM	96.64 ± 7.71	ns.
	10 nM	112.94 ± 7.91	*		10 nM	95.52 ± 7.83	ns.
	50 nM	109.72 ± 5.95	ns.		50 nM	97.97 ± 5.74	ns.
	100 nM	120.37 ± 2.36	***		100 nM	97.80 ± 2.08	ns.
	1 µM	111.08 ± 3.46	ns.		1 µM	97.55 ± 13.32	ns.
	10 µM	115.47 ± 8.49	**		10 µM	101.57 ± 4.23	ns.
	50 µM	113.44 ± 8.94	*		50 µM	104.30 ± 1.66	ns.
	100 µM	112.25 ± 3.32	ns.		100 µM	101.24 ± 5.88	ns.
**Cell line**	**Dose**	**Value ± SD [%]**	**Statistical significance**	**Cell line**	**Dose**	**Value ± SD [%]**	**Statistical significance**
SH-SY5Y	Control	100.00 ± 17.18	-	CACO-2	Control	100.00 ± 3.09	-
	1 nM	86.00 ± 2.58	ns.		1 nM	101.69 ± 7.83	ns.
	10 nM	75.51 ± 13.19	**		10 nM	102.22 ± 7.19	ns.
	50 nM	74.84 ± 8.27	***		50 nM	102.29 ± 11.18	ns.
	100 nM	71.50 ± 8.47	****		100 nM	100.68 ± 8.85	ns.
	1 µM	55.18 ± 3.12	****		1 µM	104.39 ± 1.61	ns.
	10 µM	57.97 ± 9.05	****		10 µM	103.17 ± 3.28	ns.
	50 µM	53.72 ± 4.96	****		50 µM	120.00 ± 8.46	***
	100 µM	78.60 ± 5.76	**		100 µM	131.57 ± 0.51	****

4. Les-6166

4.1. 6h-treatment

Table S10. Data with *, **, *** are statistically different from the respective (vehicle-treated) control at p<0.05, p<0.01, and p<0.001, respectively (one-way ANOVA, Tukey test), ns. – no statistical differences.

Cell line	Dose	Value ± SD [%]	Statistical significance	Cell line	Dose	Value ± SD [%]	Statistical significance
BJ	Control	100.00 ± 6.12	-	A549	Control	100.00 ± 5.20	-
	1 nM	87.20 ± 6.63	***		1 nM	118.14 ± 7.48	ns.
	10 nM	92.31 ± 5.65	ns.		10 nM	129.34 ± 7.79	***
	50 nM	98.69 ± 6.63	ns.		50 nM	122.02 ± 9.54	**
	100 nM	99.92 ± 2.63	ns.		100 nM	119.01 ± 10.59	*
	1 µM	95.70 ± 1.36	ns.		1 µM	113.21 ± 8.01	ns.
	10 µM	110.06 ± 4.02	*		10 µM	102.54 ± 19.53	ns.
	50 µM	108.14 ± 4.52	ns.		50 µM	110.97 ± 8.35	ns.
	100 µM	109.36 ± 1.73	*		100 µM	103.78 ± 2.29	ns.
**Cell line**	**Dose**	**Value ± SD [%]**	**Statistical significance**	**Cell line**	**Dose**	**Value ± SD [%]**	**Statistical significance**
SH-SY5Y	Control	100.00 ± 8.24	-	CACO-2	Control	100.00 ± 2.65	-
	1 nM	99.49 ± 7.35	ns.		1 nM	108.36 ± 10.74	ns.
	10 nM	95.84 ± 5.62	ns.		10 nM	110.50 ± 11.55	ns.
	50 nM	96.42 ± 3.05	ns.		50 nM	87.74 ± 6.93	ns.
	100 nM	93.34 ± 0.80	ns.		100 nM	93.66 ± 15.88	ns.
	1 µM	94.39 ± 3.54	ns.		1 µM	100.21 ± 7.47	ns.
	10 µM	96.65 ± 6.35	ns.		10 µM	112.11 ± 7.79	ns.
	50 µM	91.52 ± 2.69	ns.		50 µM	101.63 ± 1.29	ns.
	100 µM	98.41 ± 2.02	ns.		100 µM	84.08 ± 10.88	ns.

4.2. 24h-treatment

Table S11. Data with *, **, *** are statistically different from the respective (vehicle-treated) control at p<0.05, p<0.01, and p<0.001, respectively (one-way ANOVA, Tukey test), ns. – no statistical differences.

Cell line	Dose	Value ± SD [%]	Statistical significance	Cell line	Dose	Value ± SD [%]	Statistical significance
BJ	Control	100.00 ± 7.16	-	A549	Control	100.00 ± 6.34	-
	1 nM	101.45 ± 9.93	ns.		1 nM	113.66 ± 9.69	ns.
	10 nM	94.88 ± 7.03	ns.		10 nM	115.82 ± 2.63	*
	50 nM	114.41 ± 4.83	ns.		50 nM	113.68 ± 6.88	ns.
	100 nM	110.73 ± 2.73	ns.		100 nM	121.31 ± 13.29	***
	1 µM	111.45 ± 16.77	ns.		1 µM	123.60 ± 7.75	***
	10 µM	122.72 ± 12.78	**		10 µM	106.09 ± 6.17	ns.
	50 µM	124.53 ± 11.06	**		50 µM	105.82 ± 4.36	ns.
	100 µM	120.73 ± 1.63	*		100 µM	114.64 ± 8.13	ns.
**Cell line**	**Dose**	**Value ± SD [%]**	**Statistical significance**	**Cell line**	**Dose**	**Value ± SD [%]**	**Statistical significance**
SH-SY5Y	Control	100.00 ± 7.77	-	CACO-2	Control	100.00 ± 2.53	-
	1 nM	106.12 ± 9.71	ns.		1 nM	70.27 ± 8.98	***
	10 nM	115.27 ± 9.14	ns.		10 nM	53.25 ± 10.62	***
	50 nM	110.25 ± 5.99	ns.		50 nM	77.89 ± 14.72	**
	100 nM	121.84 ± 5.58	***		100 nM	61.98 ± 10.57	***
	1 µM	113.50 ± 4.71	ns.		1 µM	65.31 ± 10.98	***
	10 µM	115.71 ± 10.16	*		10 µM	65.97 ± 9.70	***
	50 µM	110.88 ± 5.67	ns.		50 µM	66.48 ± 9.10	***
	100 µM	114.06 ± 11.67	ns.		100 µM	64.35 ± 7.72	***

4.3. 48h-treatment

Table S12. Data with *, **, *** are statistically different from the respective (vehicle-treated) control at p<0.05, p<0.01, and p<0.001, respectively (one-way ANOVA, Tukey test), ns. – no statistical differences.

Cell line	Dose	Value ± SD [%]	Statistical significance	Cell line	Dose	Value ± SD [%]	Statistical significance
BJ	Control	100.00 ± 10.11	-	A549	Control	100.00 ± 2.36	-
	1 nM	92.24 ± 7.94	ns.		1 nM	98.09 ± 5.12	ns.
	10 nM	105.14 ± 11.94	ns.		10 nM	102.03 ± 3.71	ns.
	50 nM	120.61 ± 1.61	***		50 nM	101.77 ± 3.93	ns.
	100 nM	112.35 ± 3.73	ns.		100 nM	97.83 ± 8.14	ns.
	1 µM	113.11 ± 4.75	*		1 µM	98.12 ± 7.76	ns.
	10 µM	135.49 ± 5.80	***		10 µM	100.26 ± 1.60	ns.
	50 µM	123.56 ± 3.84	***		50 µM	99.35 ± 7.86	ns.
	100 µM	125.53 ± 1.63	***		100 µM	94.25 ± 5.50	ns.
**Cell line**	**Dose**	**Value ± SD [%]**	**Statistical significance**	**Cell line**	**Dose**	**Value ± SD [%]**	**Statistical significance**
SH-SY5Y	Control	100.00 ± 10.80	-	CACO-2	Control	100.00 ± 4.36	-
	1 nM	111.36 ± 8.91	ns.		1 nM	112.25 ± 9.63	ns.
	10 nM	116.67 ± 9.49	*		10 nM	115.84 ± 5.85	**
	50 nM	112.87 ± 5.48	ns.		50 nM	115.65 ± 8.78	**
	100 nM	112.82 ± 4.12	ns.		100 nM	112.73 ± 0.34	ns.
	1 µM	127.69 ± 6.63	***		1 µM	102.30 ± 10.11	ns.
	10 µM	127.90 ± 11.41	****		10 µM	103.99 ± 8.08	ns.
	50 µM	121.90 ± 0.62	***		50 µM	117.10 ± 4.97	**
	100 µM	129.02 ± 5.40	**		100 µM	151.99 ± 1.67	***
